# Identifying impacts of industrial co-agglomeration on carbon emissions: Evidence from China

**DOI:** 10.3389/fpubh.2023.1154729

**Published:** 2023-03-23

**Authors:** Qiong Shen, Yuxi Pan, Yanchao Feng

**Affiliations:** Business School, Zhengzhou University, Zhengzhou, China

**Keywords:** industrial co-agglomeration, urban carbon emissions, spatial spillover effect, spatial heterogeneity, cyclic accumulative effect

## Abstract

Based on panel data of 285 cities in China at the prefecture level and above from 2005 to 2020, this paper aims to study the nexus between industrial co-agglomeration and carbon emissions from dual perspectives including space and time. It adopts multiple approaches including a dynamic general method of moment, panel quantile regression model, panel threshold model, and dynamic spatial Durbin model. The non-spatial empirical results support the establishment of the threshold effect and the imbalance effect. The spatial empirical results indicate that industrial co-agglomeration poses a dramatic stimulating effect on urban carbon emissions, and its spatial spillover effect and spatial heterogeneity are conditionally established. Furthermore, heterogeneous effects are supported, such as the positive spillover effects of industrial co-agglomeration are more significant in western cities, resource-oriented cities, and non-low-carbon pilot cities. The heterogeneous influence of cost factors on industrial agglomeration and carbon emissions has also been partially confirmed. In terms of the channels and mechanism of action, the negative externalities of industrial co-agglomeration occupy a dominant position in the current status of economic development. The dynamic equilibrium between government intervention and marketization is a solid foundation for the optimization of carbon emission reduction paths.

## 1. Introduction

**Figure 1:**
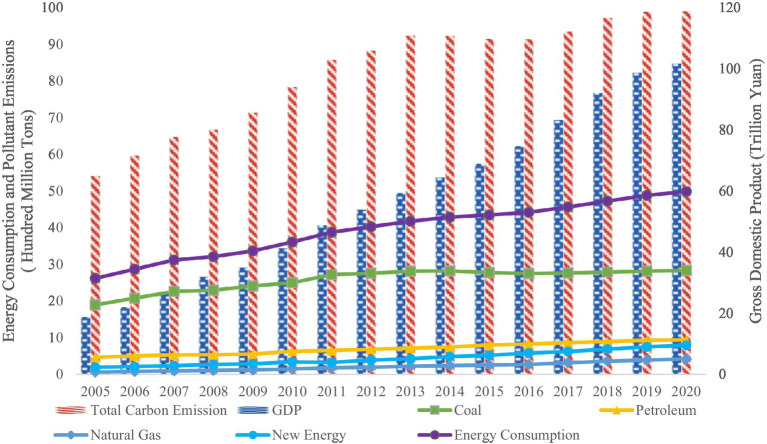
Energy consumption and pollutants levels (left scale) and economic development levels in China (right scale). Data Source: The data are collected from the International Energy Agency (https://www.iea.org/) and China Statistical Yearbook (2005–2020).

China’s economy has grown at a rapid rate since the turn of the century and has achieved great economic performance, rendering China the second-largest economy in the world, despite the reality that massive economic growth is obtained at the expense of environmental quality (Figure 1). It is evident that China’s economy showed sustained and rapid growth that requires increased total energy consumption (1). It reached the periodic peak of 49.8 hundred million tons of standard coal in 2020. The number of total carbon emissions also remained in the high-value range in recent years. As the largest source of carbon emissions, massive energy consumption and carbon emissions have resulted in a slew of issues, including major ecological and environmental imbalances (2, 3).

Based on this, the Chinese authorities have announced critical targets for energy saving and emission reduction, with China’s President Xi Jinping emphasizing the dual carbon intention of peak carbon dioxide emissions and carbon neutrality by 2030 and 2060, respectively. Under the concept of sustainable development, formulating a scientific and practical strategy has become a vital issue that needs to be solved for advancing green economic development This is also a crucial path to relieving carbon emissions and promoting low-carbon economic progress. Thereby, this paper is not only devoted to providing robust support for the achievement of Sustainable Development Goals targets (*SDGs*) but also trying to provide solid empirical experience for achieving the dual carbon target in China.

Among the methods of achieving win-win economic advancement and the alleviation of carbon emissions, the industrial co-agglomeration of the manufacturing industry and producer service industry (*IC*) receives wide attention due to the advantages such as economies of scale, competitive effects, and spillover and effects of technology (4, 5)*. IC* is an inevitable pattern for regional economic development, which refers to the interrelated industrial clusters located in a specific region. Its influences are multi-faceted and can be considered from two representative perspectives including positive externalities and negative externalities. For this reason, there are a number of issues worth exploring in depth: What are the net environmental effects of *IC*? How does *IC* affect pollutant emissions, especially urban carbon emissions (*CE*)? Does the increased degree of *IC* contribute to China’s dual carbon goal in the new phase? Against this background, the existing literature mainly pay attention to its influence on environmental quality, and the studies that have investigated the underlying impacts on *CE* can be divided into three classes. The first category of research primarily focuses on the positive externalities of *IC*, scholars recognized the contribution of *IC* to the inhibitory of environmental carbon pollution. Indeed, *IC* can improve industrial competitiveness, optimize resource allocation, and form a regional scale effect to accelerate economic growth but also can bring overflow effects of knowledge to drive improvements in energy efficiency and pose a positive impact on the quality of the ecological environment *via* technology exchange and labor cooperation (6, 7). For instance, Li and Liu (8) adopted the dynamic panel model and indicated that *IC* can significantly reduce carbon emissions through technological progress. Fang et al. (9) applied the *SBM-DEA* model to measure the carbon emission level of 282 cities in China from 2004 to 2018 and investigated the underlying influence of *IC* on carbon emissions, and their results found that collaborative agglomeration between manufacturing and producer services industries can dramatically restrain the adverse effects of industrial production on urban carbon pollutant emissions. From the views of scale effects, technological effects, and competition effects, many scholars have attempted to conduct related research (5, 10). In detail, Zhao et al. (11) conducted research from different provinces in China *via* a simultaneous equation model and proposed that the optimization of industrial structure driven by *IC* can facilitate the elimination of carbon emissions. Moreover, Wang et al. (7) adopted the system-GMM model to explore the nexus between *IC* and carbon emissions of 166 cities in China from 2005 to 2015, which concluded that the economies of scale of co-agglomeration can promote the revolution of technologies and further cut down on the number of carbon emissions.

The second category of studies held a different viewpoint based on the negative externalities of *IC*, namely it is one of the leading factors exacerbating carbon emissions that may exert damage to the quality of the environment, based on considerations of the crowding-out effect, low-end lock-in effect, rebound effect, over-competition effect, economic overcapacity, capital outflows dilemma, negative benefits of technology spillovers, and so on (12, 13). For example, Wang et al. (14) analyzed the case of the Yangtze River Delta from 2003 to 2016 to investigate the underlying effects of information and communication technology industrial agglomeration on carbon emissions and confirmed the significant positive effect of agglomeration due to the continued expansion of the economy. Shen et al. (15) proposed that *IC* may intensify to attract foreign companies to invest in highly energy-intensive and polluting industries to some extent, exacerbating the environmental pollution issues from multinational companies investing in China, and the rapid development of pollution-intensive industries exacerbates the energy consumption and pollutant emissions in the host countries. Meanwhile, the excessive co-agglomeration of regional industries can cause adverse effects on the rational allocation of regional resources and environmental carrying capacity, which is incompatible with regional pollution prevention and intensify the pressure on society to combat carbon pollution (16, 17). Hong et al. (12) built a dynamic spatial Durbin model to explore the underlying impact of *IC* on environmental pollution based on the prefecture-level cities and suggested that local government tax competition is more likely to occur the behavior of “race to the bottom” among industries, thus aggravating the negative externalities of *IC* and causing adverse effects on environmental quality.

The final category of literature has made the assertion that the nexus between *IC* and environmental pollutant emission issues is non-linear in different development stages from the diversity perspectives (18–20). For example, a typical non-linear shaped nexus between *IC* and green development (21), haze pollution (22), and industrial eco-efficiency (23) have been proposed in recent research. In addition, the multiform nonlinear relationship between *IC* and carbon emissions is also gradually being proposed and proved by scholars, such as inverted N-shaped nexus (24, 25), an inverse U-curve nexus (8, 26), a U-curve nexus (7, 20), and so on. Besides, there are a number of scholars have considered the relationship between them in depth from different perspectives. For instance, in terms of the different degrees of government intervention, Yan et al. (27) utilized the night-time light data to capture the carbon emission amount and constructed a spatial Durbin model to delve into the effect of *IC* of 268 cities in China from 2005 to 2017, confirming the double threshold effect of *IC*. As for the degree of resource mismatch, Li et al. (28) pointed out that the inhibitory effect of *IC* on urban carbon emissions is gradually weakened.

To sum up, there is undoubted that the current literature lacks a consistent viewpoint on the environmental effect of *IC* on carbon emissions, and the current state of research on this subject also leaves potential space for further improvement. First, on the basis of a constantly improving industrial structure and distribution, taking into account the underlying externalities of *IC* are complex, which may require a multi-dimensional analysis of the association between *IC* and carbon emission issues from direct, non-linear, dynamic, and spatial perspectives. Although in some of the existing studies, researchers tended to explore their possible relationship in diversified approaches (29, 30), most of them have only a single perspective, thus it is not possible to analyze the nexus between the two subjects in a systematic and integrated way. Second, the analysis of the mechanical action of *IC* is incomplete and non-objective to some extent. Even in the limited relevant studies, researchers have primarily focused on the one-sided pathway relying on the particular externality of *IC*, rather than the consideration of overall externalities. For instance, the impact channels are analyzed only from a single perspective of positive externalities (technology progress, knowledge spillover, increased energy efficiency and etc.) or negative externalities (increased energy consumption, overcapacity, excessive competition and etc.) (8, 12). And this may cause the dilemma that people subjectively choose potential paths and conduct empirical analysis based on their own baseline regression results. Third, the heterogeneity of the nexus between *IC* and urban carbon emissions should be examined in further detail. Actually, cost issues are important for the production, expansion, and spatial relocation of industries, especially the land input costs, labor input costs, and transaction costs (31, 32). Unfortunately, there have been few studies that pay attention to the effect of cost elements on the interaction between *IC* and carbon emissions, namely most studies discuss heterogeneity only in terms of traditional perspectives such as geographical location, administrative level, and resource endowment, which have ignored the reality conditions. Therefore, trying to build on and improve upon the existing research is the initial motivation for this thesis.

In this case, to make up for shortcomings of previous literature, this paper utilizes a dynamic general method of moment, panel quantile model, panel threshold model, and dynamic spatial Durbin model to investigate the effects of *IC* on carbon emissions systematically, relying on the data of 285 cities in China at the prefecture-level and above. The broad investigations in underlying channels and mechanisms of *IC* on carbon emission issues from representative positive and negative externalities are also crucial empirical content in this paper. This paper has three contributions to existing research. First, manufacturing and producer service industries are integrated into the unified analytical framework in this paper relying on the entropy method, which is in line with the frontier research directions and the reality of industrial development. This paper conducts comprehensive investigations from linear, nonlinear, spatial, and spatiotemporal perspectives, which consider the endogenous problems and robustness of estimated results. Second, this paper not only considers the heterogeneity issues from the perspectives of urban characteristics including geographical location, resource endowment, and low-carbon pilot projects but also regards the impact of cost factors on *IC* and urban carbon emissions, so as to try to help the industries find the optimal cost allocation modes. Third, this paper explores the underlying mechanisms of channels and mechanisms of action, including from the perspectives of externality, government intervention, and market regulation, which provides reference information and policy implications. Meanwhile, the empirical findings provide a new perspective for related research in academia to some extent and have far-reaching significance for the carbon reduction efforts in China and other emerging economies.

**Figure 2:**
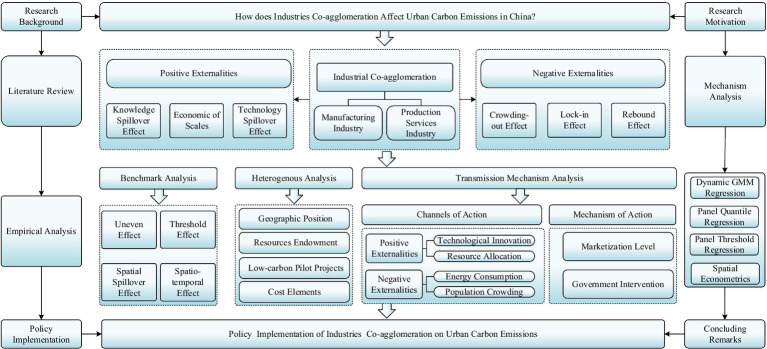
Research framework graph.

The research framework of this paper is structured as follows (Figure 2). Section 2 reveals the theoretical analysis of channels and mechanisms, while Sections 3, 4 show the methodology and core variables, respectively. Section 5 summarizes empirical results systematically. Section 6 conducts the further analysis of the heterogeneous test, channels of action, and mechanism of action. Section 7 recaps briefly and proposes policy implications.

## 2. Mechanism analysis

### 2.1. Channels of action of industrial co-agglomeration on carbon emissions

*IC* can efficiently reduce urban carbon emissions based on the positive externality theory. In terms of technological progress and innovation, the knowledge and technology overflow effect brought by *IC* is an important path to eliminating the urgency of carbon emissions (33). The high-speed development of *IC* can attract professional talent and advanced enterprises, which is conducive to forming an excellent atmosphere for innovation in a region *via* rational competition and information dissemination (10, 11). As for the allocation and utilization of resources, *IC* is beneficial to strengthen cooperation and industrial infrastructure sharing among industries, which can dramatically accelerate the flow of resource elements and improve resource allocation efficiency (34, 35). Therefore, *IC* can produce the promotion effect on industrial low-carbon transformation and seeking of a “win-win” development mode in the agglomeration area, relying on the technological innovation effect and rational resource allocation effect.

By contrast, the exacerbating effects of *IC* on carbon emissions cannot be overlooked because of negative externalities. First, the energy consumption effect is always accompanied by the development of *IC*, namely higher energy consumption, and higher pollutant emissions (36). The expansion of enterprise scale and the rebound effect of excessive co-agglomeration may result in the massive use of fossil resources and environmental carbon pollution issues (24, 37). Homogeneous imitation and excessive competition have become obvious when the *IC* degree exceeds its optimal value (38). Second, the crowding effect of *IC* also exerts a harmful influence on carbon emissions, which mainly comes from the regional heterogeneity of industry spatial layout. For instance, excessive labor agglomeration may distort the factor market and bring out a series of urgent issues, such as the congestion effect, high energy utilization, and more pollutant emissions, which is an obstacle to the coordinated development of the economy and environment (20, 39). Hence, China is still facing severe pressure on carbon emission reduction due to the adverse effects of *IC*, especially the energy consumption effect and population crowding effect.

### 2.2. Mechanism of action of industrial co-agglomeration on carbon emissions

As the “invisible hand” and “visible hand” of macroeconomic development, the market and government both yield crucial moderating effects in the achievement of carbon neutrality and carbon peak goals. From one perspective, the government can appropriately adjust the imbalance of the indusial structure and spatial pattern, which can further improve resource allocation efficiency in the market (40, 41). This is beneficial to carbon emission reduction in an indirect way. In addition, the formulation and implementation of emission reduction policies are vital guides for the low-carbon transformation of advanced enterprises. A large amount of government financial investment in environmental governance provides the necessary guarantee for sustainable innovation of green and low-carbon technologies in agglomeration areas (42). These policy-oriented actions promote the smooth progress of emission reduction directly. However, when government intervention reaches a certain threshold, it may inhibit the emission reduction effect of *IC* (27). The possible reason is that government-led industrial agglomerations may cause excessive competition among industries in pursuit of policy rent and some free-riding behaviors inhibit the reasonable allocation of resource elements (43, 44).

**Figure 3:**
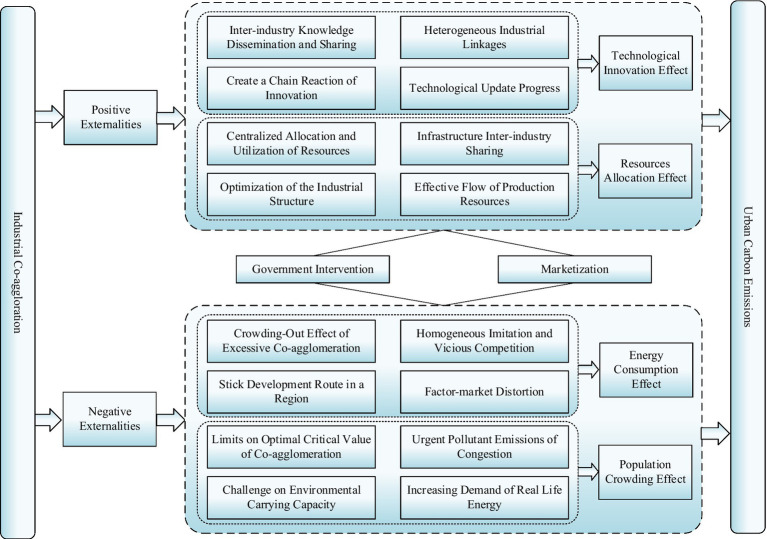
Channel and mechanism graph.

From another perspective, marketization can also be viewed as a crucial path to regulating the nexus between *IC* and carbon emissions. A rational and complete market can provide a suitable production environment for industries that promotes expansion at an industrial scale, posing a significant positive influence on regional green innovation, relying on the economy of scale effect (45). The establishment of a unified carbon emission trading market and the promotion of energy conservation and emission reduction through market mechanisms have become vital points of high-quality economic expansion. Moreover, the marketization degree is also associated with government actions. For instance, the government pays more attention to economic expansion at lower marketization levels, which tends to take the rapid development of *IC* as the priority and neglects environmental protection (46). Thereby, the sustainable development of *IC* and the achievement of the double carbon target needs to reflect the dynamic balance between the government and the market. The underlying channels and mechanism of *IC* on the *CE* in this paper can be represented in Figure 3.

## 3. Empirical methodology

### 3.1. Non-spatial econometric methods

To further investigate the influences and mechanisms of *IC* on carbon emissions, this paper adopts the pooled ordinary least squares (*OLS*), the dynamic general method of moment (*GMM*), and other non-spatial transitional regression models. First, because of the dynamic effect and inertia effect of the environmental tolerance for carbon emissions, the dynamic *GMM* that introduces the lagged-explained variable into model consideration is utilized and a pooled *OLS* is performed. The baseline regressions are constructed as in Equations 1 and 2.


(1) $$CE_{it}=\beta_{0}+\beta_{1}IC_{it}+\theta X_{it}+\varepsilon_{it}$$

(2) $$CE_{it}=\beta_{0}+\varphi CE_{i,t-1}+\beta_{1}IC_{it}+\theta X_{it}+\varepsilon_{it}$$

where *CE_it_* stands for carbon emissions in city *i* at year *t*, which is represented and measured by two core variables *TCE* and *CEI*. *IC_it_* denotes the industrial co-agglomeration level in city *i* at year *t*; *φ* represents the time response coefficient. *β*_1_ shows the estimation coefficient of *IC* and *β_0_* stands for the constant term. A series of control variables are represented by *X_it_*; *ε_it_* means the random perturbation term.

Second, in consideration of the distinct environmental inclusion and carbon emission levels among sample cities, the panel quantile regression approach was employed to identify the heterogeneity of *IC* and other influence factors on *CE* at different quantiles, and the empirical model is set as shown in Equation 3.


(3) $$CE_{it}=\beta_{0\tau }+\beta_{\tau }IC_{it}+\theta_{\tau }X_{it}+\varepsilon_{it}$$

where *τ* stands for the corresponding quantiles. The meanings of the other parameters are consistent with Equation 1.

Third, to determine whether the non-linear effect of *IC* on *CE* exists, this paper adopts a panel threshold model and introduces gross domestic production *per capita* (*PGDP*) as the suitable threshold variable to divide the constructed model into disparate intervals under the unbalanced economic development degrees. The empirical model is structured as shown in Equation 4.


(4) $$CE_{it}=\beta_{0}+\beta_{1}IC_{it}\cdot I\left(q_{it}\leq \eta \right)+\beta_{2}IC_{it}\cdot I\left(q_{it}>\eta \right)+\theta X_{it}+\varepsilon_{it}$$

where *q_it_* stands for the threshold variable and *η* means the threshold value. *I (·) i*s an instruction function.

### 3.2. Spatial econometric methods

Considering that IC can be regarded as a vital phenomenon of modern economic activities, co-agglomeration degree, and the spatial pattern are constantly changing in the dynamic accumulation process. This paper utilizes static and dynamic spatial Durbin models (*SDM*) to further investigate the temporal, spatial, and spatio-temporal effects of *IC* on carbon emissions. Moreover, a range of spatial applicability tests is carried out in turn to determine the optimal regression model. The statistical results of the Lagrange multiplier test (*LM*) and likelihood ratio test (*LR*) all pass the significance test. So, the *SDM* cannot degenerate into the spatial autoregressive model or spatial error model. Sequentially, combining the results of the Hausman statistic and joint significant test, *SDM* with dual-fixed effects is the optimal choice in this paper. Referring to the research of Elhorst (47), the equations are shown in Equations 5 and 6.


(5) $$\begin{array}{l} CE_{it}=\rho \sum_{i\neq j}^{n}W_{ij}CE_{it}+\beta_{1}IC_{it}+\varphi \sum_{i\neq j}^{n}W_{ij}IC_{it}+\\ \beta_{2}X_{it}+\theta \sum_{i\neq j}^{n}W_{ij}X_{it}+\mu_{i}+v_{t}+\varepsilon_{it} \end{array}$$

(6) $$\begin{array}{l} CE_{it}=\phi CE_{it-1}+\rho \sum_{i\neq j}^{n}W_{ij}CE_{it}+\beta_{1}IC_{it}+\varphi \sum_{i\neq j}^{n}W_{ij}IC_{it}+\\ \beta_{2}X_{it}+\theta \sum_{i\neq j}^{n}W_{ij}X_{it}+\mu_{i}+\nu_{t}+\varepsilon_{it} \end{array}$$

where *i* and *j* stand for urban regions and *t* represents the year. The spatial effect of *CE* in the local region on its surrounding areas is described by *ρ*, namely the spatial auto-regressive parameter; *φ* measures the spatial interaction of *CE* for the *IC* level in adjacent areas. *β*_1_ and *β*_2_ are the general regression coefficients, *and μ_i_* and *v_t_* exemplify the time and city-fixed effects, respectively.

This paper utilizes the geographic inverse distance matrix to measure the interactions between distant spatial units, which is more in line with the reality of the spatial associations between sample regions. The definition of the spatial weighted matrix is shown in Equation 7, and this paper also normalizes the geographic distance matrix in the empirical process:


(7) $$w_{ij}=\{\begin{array}{l} \frac{1}{{\left(d_{ij}\right)}^{2}},\;\;i\neq j\;\\ 0,\;i=j \end{array}$$

where *d_ij_* refers to the straight-line geographic distance from city *i* to city *j*, which is calculated by latitude and longitude coordinates.

## 4. Data and variables

### 4.1. Variable descriptions

#### 4.1.1. Dependent variable: Carbon emissions

The manufacturing and production services industries can be identified as two dominant sources of national carbon dioxide emissions in China, which have become the key issues in achieving scientific and precise reduction targets of carbon emissions during the 14th Five-Year Plan period. Considering the differences in carbon emission levels in different regions and the top-level design of double control, this paper selects total carbon emissions (*TCE*) and carbon emission intensity (*CEI*) to quantify the *CE* degree. Regarding the research of Zhang et al. (48), *TCE* is measured by the sum of carbon emissions from three typical energy, including natural gas, electricity, and liquefied petroleum gas in a region over a period. The detailed calculation formula is Equation 8:


(8) $$TCE=\lambda_{1}NGas+\lambda_{2}LPGas+\lambda_{3}\left(\kappa \cdot Elec\right)$$

where $\lambda_{1}$, $\lambda_{2}$ and $\lambda_{3}$ refer to the carbon emission factor for natural gas, liquefied petroleum gas, and electricity respectively, which are equal to 2.1622 (kg/m^3^), 3.1013 (kg/kg), and 1.3023 (kg/kW h). Variable κ stands for the proportion of coal-fired electricity generation.

#### 4.1.2. Core independent variable: Industrial co-agglomeration

From the contribution of Yang et al. (36), this paper utilizes the location entropy approach to evaluate the status of IC from two special perspectives mentioned above. The specific formulas are shown as Equations 9 and 10:


(9) $$IA_{ij}=\frac{Q_{ij}/Q_{j}}{Q_{i}/Q}$$

(10) $$IC=1-\frac{\left|IA_{MFI}-IA_{PSI}\right|}{\left(IA_{MFI}+IA_{PSI}\right)}$$

where *IA_ij_* shows the measurement of agglomerations of the manufacturing industry (MFI) or producer service industry (PSI) in city *j*. *Q_ij_* represents the number of laborers in industry *i* in sample city *j*. *Q_j_* indicates the number of members of the two types of industries in city *j*. *Q_i_* stands for the sum of people employed by industry *i* in the whole country. *Q* is the overall number of laborers of the two target industries mentioned above. The rest of the symbols are the same as described above.

#### 4.1.3. Control variables

Apart from key explanatory variables, other underlying variables are considered controls. According to the literature (49–51), the selected control variables consist of fiscal decentralization (*FD*), industrial upgrading (*IU*), urbanization rate (*UR*), and foreign direct investment (*FDI*). *FD* is measured by the proportion of financial expenditure to financial revenue. *IU* is measured by the proportion of the added value of the tertiary industry to the secondary industry. *UR* is measured by the ratio of non-farm population to population size in a city. *FDI* is measured by the ratio of foreign direct investment to gross domestic product.

#### 4.1.4. Threshold variable

In agreement with the current study of Feng and He (49), this paper chooses *PGDP* as a threshold variable for further investigations at various economic levels. This indicator has been expressed by the real *PGDP* which takes the year 2005 as the base period and is logarithmically processed to assure the comparability and consistency of the data.

#### 4.1.5. Mechanism variables

Following previous studies (52–54), a series of mechanism variables are expressed normatively. Technological innovation (*TI*) is expressed by the number of patent applications per 10,000 people. Resource allocation (*RA*) is expressed by total factor productivity through the *SFA* method, which uses the number of employees and fixed assets as input with real *GDP* as output. Energy consumption (*EC*) is indirectly expressed by urban consumption level, namely total retail sales of consumer goods in a city are adopted as a reasonable proxy. Population crowding (*PC*) is expressed by the number of populations at year-end in a city. Government intervention (*GI*) is expressed by the ratio of fiscal expenditure to real gross domestic product. Marketization level (*ML*) is expressed by the Fan Gang index of market liberalization in a city.

#### 4.1.6. Instrumental variables

Regarding the existing literature (55, 56), the proxies related to infrastructure construction can be regarded as reasonable instrumental variables (*IV*), which satisfy the validity principles of *IV* selection. First, the public product attribute and investment characteristics of infrastructure are directly under the macro-control of the government, independent of the urban pollutant emissions, which satisfies the exogenous requirement of an instrumental variable. Second, infrastructure is a key resource for regional economic and social development, and it can also exert potential influences on regional industrial spatial layout, which satisfies the correlation requirement.

Therefore, this paper adopts the construction of highway mileage per unit area (*IV_1_*) and the interaction term of the number of post stations in the Ming dynasty and the number of taxis during the current period (*IV_2_*) as the instrumental variables of *TCE* and *CEI*, separately. The reason for the utilization of the interaction term is that the number of post-stations in the Ming dynasty is a fixed value that cannot be directly plugged into the regression model. The final construction of the interaction term is based on Nunn and Qian (57) and the consideration of post-stations’ functional features.

Table 1 reports the depictions and descriptive statistics of core variables. The obtained data are all within the rational distribution interval, which shows the accuracy and authenticity of data values.

**Table 1:** Depictions of core variables and descriptive statistics.

Variables	Symbols	Depictions	Unit	Obs.	Mean	SD	Min	Max
Dependent variable								
*Total carbon emissions*	*TCE*	The total greenhouse gas emissions of a region or city over a certain period	Million tons	4,560	27.461	24.178	1.723	230.712
*Carbon emission intensity*	*CEI*	Total carbon emissions/Real GDP *Per Capita*	/	4,560	8.216	6.693	0.446	90.571
Core independent variable								
*Industrial co-agglomeration*	*IC*	Refers to co-agglomeration degree of manufacturing industries and production service industries	/	4,560	0.735	0.185	0.066	0.999
Control variables								
*Fiscal decentralization*	*FD*	Financial expenditure/Financial revenue	/	4,560	2.868	1.928	0.649	18.399
*Industrial upgrading*	*IU*	Add value of tertiary industry/The secondary industry	%	4,560	0.946	0.529	0.094	5.348
*Urbanization rate*	*UR*	Non-farm population/Total population in a city	%	4,560	0.510	0.168	0.112	1.000
*Foreign direct investment*	*FDI*	Foreign direct investment/Gross domestic product	%	4,560	0.021	0.023	0.000	0.285
Threshold variable								
*GDP per capita*	*PGDP*	Gross domestic product achieved in a country in one year/country’s resident population	CNY	4,560	9.638	0.658	7.782	11.835
Mechanism variables								
*Technological innovation*	*TI*	Patent applications numbers per ten thousand people	/	4,560	8.324	21.944	0.011	651.563
*Resource allocation*	*RA*	Total factor productivity	/	4,560	1.436	0.767	0.020	2.948
*Energy consumption*	*EC*	Total retail sales of consumer goods in a city	CNY	4,560	15.186	1.179	5.472	18.886
*Population crowding*	*PC*	Population at the year-end in a city	Ten thousand people	4,560	5.867	0.697	2.846	8.136
*Government intervention*	*GI*	Fiscal expenditure/ Gross domestic product	/	4,560	0.180	0.105	0.043	1.936
*Marketization level*	*ML*	Fan Gang index of market liberalization in a city	/	4,560	10.384	2.908	2.717	19.694
Instrumental variables								
*Infrastructures-IV_1_*	*IV_1_*	The construction of highway mileage per unit area	Km	4,560	0.992	0.503	0.030	2.628
*Infrastructures-IV_2_*	*IV_2_*	The interact term of the number of post stations in the Ming dynasty and the number of taxis at current period	/	4,560	14.042	62.839	0.000	898.500

### 4.2. Data collection

The panel dataset of 285 Chinese cities at the prefecture level and above from 2005 to 2020 are collected as sample data, which are obtained from official statistical publications including China City Statistics Yearbook (2005–2020), China Statistical Yearbook (2005–2020) and China Urban Construction Statistical Yearbook (2005–2020). All obtained data have been reconfirmed to ensure accuracy and reliability. Some missing data have been supplemented by manual queries, and only a small number of the missing values was calculated by the interpolation approach. The natural logarithm is applied to the data of *PGDP*.

## 5. Empirical estimates results

### 5.1. Empirical analysis of non-spatial baseline regressions

Based on pooled *OLS* regression and dynamic *GMM* approaches, the interactions of *IC* on carbon emissions in current years are evaluated and confirmed. The results are reported in Table 2. The coefficients of the first-order lagged term of the carbon emissions proxies are both significantly positive at a 1% confidence interval, which demonstrates that the current carbon emissions degree is significantly stimulated by the historical carbon emission levels and the temporal inertia characteristics of China’s carbon emission evolution are verified. Energy conservation and emission reduction are of long-term significance and are conducive to fostering high-quality and sustainable economic growth in the future. The estimated results of different models yield the consistent conclusion that *IC* can intensify regional carbon emissions, including *TCE* and *CEI* to some extent, which means higher *IC* may produce malignant impacts on the ecological environment. The results are consistent with the conclusion obtained by Hong et al. (12), confirming that the crowding effect and “race-to-bottom” competition caused by excessive agglomeration is one of the important reasons for aggravating local environmental pollution. Furthermore, the *AR* test verifies the evidence of the first-order correlation in the residual series and no second-order correlation exists. The value of ps of the Sargan test are both equal to zero, representing that the selected instrumental variables in estimated models are not highly exogenous and suffer from over-reorganization restrictions. Therefore, given the limitations of the above regression methods and the accuracy of estimations, the instrumental variable strategy of the endogeneity test is involved in the subsequent part.

**Table 2:** Estimation results of benchmark regression and dynamic GMM.

Core variables	*OLS*		*SYS-GMM*	
	*TCE*	*CEI*	*TCE*	*CEI*
	(1)	(2)	(3)	(4)
*L.TCE*			1.041***	
			(292.967)	
*L.CEI*				0.941***
				(254.091)
*IC*	0.462	0.917**	2.109***	0.220
	(0.666)	(2.515)	(4.457)	(1.194)
*FD*	−0.080	0.250***	0.188***	−0.072***
	(−0.897)	(5.326)	(3.376)	(−3.400)
*IU*	11.523***	−3.617***	−9.969***	2.263***
	(8.275)	(−4.949)	(−9.892)	(9.499)
*UR*	−0.013	0.831***	−1.424***	0.334***
	(−0.041)	(4.897)	(−9.544)	(6.670)
*FDI*	−19.566***	−21.378***	7.882***	2.377***
	(−3.926)	(−8.173)	(3.086)	(3.078)
Constant	13.500***	13.142***	3.776***	−1.556***
	(14.496)	(26.887)	(7.245)	(−7.147)
Observations	4,560	4,560	4,275	4,275
Number of cities	285	285	285	285
*R*-squared	0.502	0.547		
AR (1)			0.002	0.000
AR (2)			0.245	0.506
Sargan test			0.000	0.000

***p < 0.01, **p < 0.05, *p < 0.1. *T*-values in parentheses of OLS, Z-statistics in parentheses of GMM, the results of AR (1) and AR (2) statistics, and Sargan test are the corresponding *p*-value.

According to the above empirical analysis, it is obvious that the results of pooled *OLS* and dynamic *GMM* are somewhat differentiated, especially the control variables, so this paper also conducts the endogeneity test to avoid potential biases from endogenous problems. The two-stage least square (*2SLS*) approach is adopted with the above-selected instrumental variables, which satisfy the two reasonable requirements to further analyze the baseline results. As shown in Table 3, the *F*-value of *IV*_1_ and *IV*_2_ in first-stage regression both exceed 10, reflecting the selected instrumental variables are highly correlated with explanatory variables and that there are no biases from weak instrumental variables. The estimations in second-stage regression in columns (2) and (4) show that the coefficients of *IC* are consistent with the original results in baseline regressions, namely, *IC* still contributes to the carbon emissions even considering the endogeneity of core variables, which indicates the robustness of the obtained conclusions. Therefore, this paper adopts a series of transitional regression models to evaluate the effects of *IC*.

**Table 3:** The results of endogeneity test.

	Endogeneity test for *TCE*		Endogeneity test for *CEI*	
	(1)	(2)	(3)	(4)
	*IC*	*TCE*	*IC*	*CEI*
*IV_1_*	0.045***			
	(0.013)			
*IV_2_*			−0.000***	
			(0.000)	
*IC*		7.567		304.899***
		(15.272)		(103.287)
First-stage *F* statistic value	12.607		12.247	
Controls	Yes	Yes	Yes	Yes
City Fixed Effect	Yes	Yes	Yes	Yes
Year Fixed Effect	Yes	Yes	Yes	Yes
Number of cities	285	285	285	285
Observations	4,560	4,560	4,560	4,560
*R*-squared	0.713	0.965	0.712	-

***p < 0.01, **p < 0.05, *p < 0.1. Robust standard errors in parentheses.

The results of the panel quantile regression framework with the consideration of distributional heterogeneity indicate the aggravated and uneven influences of *IC* on carbon emissions. The intensification effect of *IC* on *TCE* shows an increasing fluctuation trend with the increasing emission levels, nevertheless, the promotion effect of *IC* on *CEI* is characterized by continuous and stable increases. Furthermore, the threshold effects of *IC* on carbon emissions are verified by the estimations of panel threshold regression, including the single threshold model of *TCE* and the double threshold model of *CEI*. In terms of *TCE*, *IC* plays a significant negative role in *TCE* when the level of *PGDP* is below the value of 10.3, nonetheless, its emission-reduction effect is weakened. It shifts to positive if the *PGDP* level exceeds this threshold value. This result is similar to the research of (58), which supports that *IC* may be beneficial to environmental quality in the initial stage, but with the expansion of economic scale and the improvement of agglomeration degree, industrial agglomeration may cause damage to environmental quality. By contrast, the *IC* has a persistent aggravated effect on *CEI* before the *PGDP* level reaches the second threshold value of 9.4, and this positive effect reverses and becomes less significant. Hence, with China’s rapid industrial economic development, the evolution of *TCE* and *CEI* is not consistent, so there is still a long way to go to achieve the targets of the dual-carbon goal. The specific tables and figures are reported in the Supplementary material.

### 5.2. Empirical analysis of spatial baseline regressions

As for the spatial correlation test, this paper chooses Moran’s *I* and Geary’s *C* to reflect the global spatial distribution pattern and local spatial autocorrelation, correspondingly. Moran’s *I* and Geary’s *C* in 2005–2020 all pass the significance test at a 1% confidence level, which verifies the validity of spatial econometric models. According to the crucial scatterplots of Moran’s *I*, the spatial distribution patterns of carbon emissions in China in the majority of cities are distributed in the first and third quadrants (H-H and L-L aggregation zones), which provides evidence for the positive spatial autocorrelations. The table of spatial autocorrelation tests and crucial scatterplots is in the Supplementary material.

Comparing the results of *SDM* and dynamic *SDM*, it can be found that the obtained highlights are still valid when dynamic factors are considered. This paper focuses on the analysis of dynamic *SDM*. The results of static *SDM* are in the Supplementary material.

According to Tables 4, 5, the following key points can be concluded. First, the positive spatial overflow effects of carbon emissions in China are due to the significance of spatial autoregressive coefficients. Second, the coefficients of the two measurement indexes at a one-period spatial lag are all significantly positive at a 1% confidence interval, which demonstrates urban carbon emissions in China are a continuous dynamic adjustment process and showed dynamic and spatio-temporal effects. The dynamic effects of carbon emissions in China are relatively limited because of the high consistency of directionality and significance of coefficients in the two spatial models. The long-term equilibrium between high-quality economic growth and carbon emission control is persistent, which reveals that the realization of China’s dual carbon goal faces great pressure. The probable reason for this phenomenon is that the expansion of industrial scale and economic growth brought by *IC* can lead to a rapid increase in energy consumption, which causes the aggravation of carbon emissions. Moreover, limited resources in the region may make vicious imitation and excessive competition among enterprises and hinder the development and application of new technologies, which may also pose adverse shocks to the environmental capacity of the region. Third, based on the coefficients of effect decomposition, *IC* exerts an intensifying effect on *TCE* in surrounding regions and the whole society, and the accumulating effect is supported. *IC* also poses an accelerating effect on *CEI* in a region, but this stimulatory effect is diminishing. This conclusion of the negative spatial spillover effect of *IC* on carbon emissions is similar to the works of Cheng (16). It reveals the essence of the “race to the bottom,” the competition among local governments may lead to serious industrial homogenization in agglomerations, and the attraction of the low-level industries may further solidify the traditional extensive development mode of the agglomerations, which both are the main paths to increase the carbon emission in adjacent regions (59). Finally, the differentiated effects of control variables can be observed, which reveals the complexity of the economic impacts on the formulation of carbon emissions policy. The challenges in achieving the transition from peak carbon to carbon-neutral are enormous. These empirical results provide evidence for the negative influence of beggar-thy-neighbor practice, which reveals policy actions need to be further adjusted. The government has strengthened environmental regulations to seek the dual carbon target and the promotion of energy conservation and emission reduction. This has guided the gradual transformation of industrial agglomeration regions toward green development, and the accelerating effect tends to be alleviated in the long term.

**Table 4:** Results of dynamic SDM with dual-fixed effects (*TCE*).

Core Variables	Regression		Short-term			Long-term		
	Main	Weight matrix	Direct effect	Indirect effect	Total effect	Direct effect	Indirect effect	Total effect
	(1)	(2)	(3)	(4)	(5)	(6)	(7)	(8)
*L.WTCE*	0.418***							
	(14.743)							
*IC*	−0.065	2.487***	0.018	2.726***	2.743**	0.527	4.716**	5.243**
	(−0.108)	(2.754)	(0.030)	(2.687)	(2.301)	(0.773)	(2.566)	(2.292)
*FD*	−0.240***	0.452***	−0.214***	0.454***	0.240*	−0.152*	0.611***	0.459*
	(−2.872)	(3.436)	(−2.728)	(3.360)	(1.791)	(−1.840)	(2.753)	(1.794)
*IU*	−0.874***	1.137**	−0.834***	1.173**	0.340	−0.703**	1.354*	0.651
	(−2.953)	(2.542)	(−2.959)	(2.531)	(0.684)	(−2.287)	(1.707)	(0.684)
*UR*	10.434***	−3.816**	10.344***	−2.691	7.652***	10.934***	3.703	14.637***
	(7.935)	(−2.074)	(8.203)	(−1.329)	(3.572)	(8.024)	(1.062)	(3.518)
*FDI*	−19.386***	25.468***	−18.149***	25.033***	6.884	−15.406***	28.536**	13.130
	(−4.162)	(3.895)	(−4.016)	(3.623)	(0.981)	(−3.231)	(2.507)	(0.976)
*ρ*		0.123***						
		(5.370)						
Observations	4,275	4,275	4,275	4,275	4,275	4,275	4,275	4,275
*R*-squared	0.531	0.531	0.531	0.531	0.531	0.531	0.531	0.531
Number of cities	285	285	285	285	285	285	285	285

*p < 0.1; **p < 0.05; ***p < 0.01. *Z*-values in parentheses.

**Table 5:** Results of dynamic SDM with dual-fixed effects (*CEI*).

Core variables	Regression		Short-term			Long-term		
	Main	Weight matrix	Direct effect	Indirect effect	Total effect	Direct effect	Indirect effect	Total effect
	(1)	(2)	(3)	(4)	(5)	(6)	(7)	(8)
*L. WCEI*	0.324***							
	(10.643)							
*IC*	1.481***	−0.910*	1.443***	−0.824	0.619	1.418***	−0.454	0.963
	(4.553)	(−1.875)	(4.601)	(−1.552)	(1.000)	(4.184)	(−0.585)	(0.998)
*FD*	0.082*	0.269***	0.095**	0.291***	0.386***	0.138***	0.463***	0.600***
	(1.822)	(3.793)	(2.251)	(4.041)	(5.454)	(3.233)	(4.675)	(5.389)
*IU*	0.691***	−0.181	0.684***	−0.102	0.583**	0.709***	0.198	0.907**
	(4.343)	(−0.755)	(4.505)	(−0.415)	(2.265)	(4.538)	(0.580)	(2.260)
*UR*	−1.611**	−2.310**	−1.681**	−2.580**	−4.261***	−2.107***	−4.526***	−6.632***
	(−2.271)	(−2.309)	(−2.446)	(−2.470)	(−3.802)	(−2.967)	(−3.095)	(−3.783)
*FDI*	−9.933***	−13.417***	−10.269***	−15.490***	−25.759***	−12.836***	−27.276***	−40.112***
	(−3.961)	(−3.786)	(−4.198)	(−4.094)	(−6.685)	(−5.156)	(−5.090)	(−6.470)
*ρ*		0.092***						
		(3.925)						
Observations	4,275	4,275	4,275	4,275	4,275	4,275	4,275	4,275
*R*-squared	0.519	0.519	0.519	0.519	0.519	0.519	0.519	0.519
Number of cities	285	285	285	285	285	285	285	285

*p < 0.1; **p < 0.05; ***p < 0.01. *Z*-values in parentheses.

To assess the validity of the empirical results, this paper adopts the robustness tests from diversified perspectives, including replacing explained variables, replacing research samples, and constructing new spatial weight matrices. The re-regression estimations illustrate that the core coefficients are in the same direction, and there are only some changes in significance levels, validating the robustness of benchmark estimations. The specific process and empirical results of the robustness test are presented in Supplementary materials.

## 6. Further analysis

### 6.1. Further analysis of heterogeneous effect

This section explores the heterogeneous effects of *IC* on carbon emissions, which classifies the heterogeneities from the perspectives of geographic position, resource endowment, and low-carbon pilot projects. This part addresses the heterogeneous effect of *IC* on *TCE*, and the results are shown in Table 6.

**Table 6:** Results of heterogeneity test.

Effects	Core variables	Geographical location			Resources endowment		Low carbon pilot projects	
		Eastern	Central	Western	Resource-oriented	Non-resource-oriented	Pilot Cities	Non-pilot Cities
		(1)	(2)	(3)	(4)	(5)	(6)	(7)
Spatial effect	*ρ*	0.047	0.149***	0.080*	0.073**	0.121***	0.054	0.155***
		(1.210)	(4.126)	(1.848)	(2.194)	(4.146)	(1.153)	(6.134)
Direct effect	Short-term	2.862**	0.561	−3.320***	1.232*	−0.102	−4.859**	1.162**
		(2.342)	(0.680)	(−3.322)	(1.764)	(−0.114)	(−2.303)	(2.446)
	Long-term	2.291*	1.248	−2.310*	1.839**	−0.138	−4.819**	1.473***
		(1.703)	(1.288)	(−1.894)	(2.159)	(−0.138)	(−2.286)	(2.595)
Indirect effect	Short-term	−4.530**	3.450**	5.926***	1.657	−0.208	−4.830	0.912
		(−2.308)	(2.482)	(3.636)	(1.628)	(−0.133)	(−1.216)	(1.095)
	Long-term	−5.159*	6.220**	7.607**	3.865**	−0.362	−4.648	2.591*
		(−1.672)	(2.494)	(2.471)	(2.058)	(−0.152)	(−1.188)	(1.658)
–	Observations	1,515	1,635	1,125	1,725	2,550	1,020	3,255
–	*R*-squared	0.485	0.550	0.592	0.565	0.502	0.164	0.628
–	Number of cities	101	109	75	115	170	68	217

***p < 0.01, **p < 0.05, *p < 0.1. *Z*-values in parentheses.

#### 6.1.1. Heterogeneity in geographic position

Considering cities’ unique characteristics in different geographical regions, this paper splits samples into eastern, central, and western cities. The results in columns (1)–(3) show the effect of *IC* on urban *CE* in different areas is different. In terms of the effect decomposition, *IC* in eastern cities plays a significant positive role in local carbon emissions and this intensification effect decreases over time; nevertheless, *IC* poses a negative spatial overflow effect on carbon emissions of neighboring cities. The opposite effects in western cities are significant. This is mainly because the high level of economic growth in the east attracts abundant industries and laborers moving from underdeveloped regions and may result in population concentration. Although overpopulation pressures have brought about a series of passive influences in a region, the interregional movement of production elements can reduce environmental regulation stress in the surrounding areas. By contrast, the ecological improvement and environmental protection in the western area promote the agglomeration of clean energy industries and increase energy efficiency. Moreover, high-consuming and high-polluting industries tend to move to peripheral cities which may intensify the *CE* levels in non-central areas due to the unbalanced development.

#### 6.1.2. Heterogeneity in resource endowment

Considering the specific resource endowment of different cities, this paper differentiates samples into resource-oriented and non-resource-oriented cities shown in columns (4, 5). The accelerating effect and accumulative effect of *IC* in resource-oriented cities are significant. In practice, the development of resource-based cities relies on their resource endowment, which generally ignores the accumulation and management of fundamental production factors such as high-tech, talents, and capital. The unscientific development model and unreasonable industrial structure may aggravate energy consumption and reduce the efficiency of resource allocation, which can aggravate local carbon emissions levels. The negative externalities of the path-dependence effect and vicious competition have progressively occupied a dominant position, which also limits the knowledge and technology spillover effects to neighboring areas.

#### 6.1.3. Heterogeneity in low-carbon pilot projects

Considering the potential influence of the national macro-policy, this paper classifies samples into low-carbon pilot cities and non-pilot cities and presents results in columns (6, 7). It can be observed that the *IC* in low-carbon pilot cities can restrain local carbon emissions and tend to release the carbon emission pressure in neighboring regions, nonetheless, *IC* exerts the opposite effect in non-pilot cities. This may be because the low-carbon pilot cities adhere to the development concepts of resource conservation and environment-friendliness, which promotes the transformation of industries to low-carbon development and dramatically reduces carbon emissions. Low-carbon pilot cities are a model for other regions and cities.

#### 6.1.4. Heterogeneity in cost elements

Considering the reality that different cities have different economic features and different combined cost characteristics, which would impose distinct restrictions on the development of *IC* and affect its associations with urban carbon emission issues. Indeed, the land price is a key factor in determining the optimal size of industrial operations and the basis for the stochastic relocation and expansion of agglomeration. Meanwhile, based on the “industrial location theory” first proposed by the German economist (60), it emphasized that transport costs and labor costs are crucial factors influencing the choice of industrial location. In this case, this paper tends to explore the role of heterogeneity due to cost factors and classifies the sample cities into two groups, namely a high-level group and a low-level group, based on the average level of the cost elements in 2020. In detail, labor costs are represented by the regional average wage level of urban workers. Land costs are represented by regional average property prices. Transaction costs are represented by the cost of transport conditions that equals the ratio of road mileage to urban land area, and it is an inverse indicator, namely the higher the value, the lower the cost.

The results of heterogeneous tests are shown in Table 7, the heterogeneity is relatively pronounced. First, in terms of labor costs, it can be found that *IC* has a certain tendency to inhibit *CE* in the regions with higher labor costs according to columns (1, 2), which is opposite to the area with lower labor costs. The possible reason may lie in the fact that the higher labor costs illustrate the higher salary in a region, which can increase labor mobility and attract a concentration of talent, so as to promote the technological innovation and progress of enterprises relying on the knowledge spillover and technology sharing effects. Meanwhile, the high labor costs may encourage industries to spend more capital and resources on the introduction of high-tech talent, which is a vital force for technological green transformation in enterprises and pollution control in agglomerations (61). By contrast, the labor-intensive industries with high pollution and high-emission features tend to move into areas with low labor costs, which brings challenges to environmental quality in these areas. Second, as for the land costs in columns (3, 4), *IC* in areas with high land costs can significantly intensify local *CE*, nonetheless, it has an inhibitory effect on surrounding areas with low labor costs. This is mainly because the higher land cost causes limited industrial development space, which is difficult for enterprises to have enough financial support to realize high-quality green transformation and upgrading. Meanwhile, they also need to expand their production scale to increase profits and compensate for the high costs of investment, namely, overcapacity is an elemental contributor to increased carbon emissions (62). Third, in terms of transaction costs, heterogeneity between cities with different transaction costs is not obvious, the positive spatial overflow effect of *IC* is relatively significant in high-cost regions, this phenomenon may rely on the industry’s consideration of the convenience of the production chain and the fact of industrial transfer. Thereby, the heterogeneity in cost elements is partially established, especially reflecting the role of labor and land costs, and the crucial significance of reasonable cost management and control in promoting healthy and collaborative industry development.

**Table 7:** Results of heterogeneity test in cost elements.

Effects	Core variables	Labor costs		Land costs		Transaction costs	
		High-level	Low-level	High-level	Low-level	High-level	Low-level
		(1)	(2)	(3)	(4)	(5)	(6)
Spatial effect	*ρ*	0.087***	0.114***	0.061	0.163***	0.151***	0.064*
		(2.765)	(3.525)	(1.451)	(6.480)	(5.192)	(1.878)
Direct effect	Short-term	−0.950	0.782	3.601**	−0.295	0.260	0.224
		(−0.759)	(1.602)	(2.168)	(−0.611)	(0.352)	(0.240)
	Long-term	−1.218	1.061*	2.377	0.069	1.070	0.362
		(−0.853)	(1.784)	(1.273)	(0.110)	(1.060)	(0.381)
Indirect effect	Short-term	−1.242	0.788	−9.501***	1.604**	2.751**	1.656
		(−0.596)	(0.875)	(−3.377)	(1.970)	(2.162)	(1.104)
	Long-term	−2.242	2.420	−12.016***	2.917	5.863**	2.087
		(−0.721)	(1.272)	(−2.728)	(1.627)	(2.029)	(1.143)
–	Observations	1,905	2,370	1,290	2,985	2,190	2,085
–	*R*-squared	0.256	0.301	0.246	0.381	0.394	0.153
–	Number of cities	127	158	86	199	146	139

***p < 0.01, **p < 0.05, *p < 0.1. *Z*-values in parentheses.

### 6.2. Further analysis in channels of action

The empirical results provide vigorous evidence that *IC* can dramatically stimulate *CE*, reflecting the challenges of achieving a dual carbon target and the urgency of carbon emission governance. Further considerations of the underlying channels are indispensable. Based on this insight, regression Equation 11 is constructed to analyze the channels of action. The results are reported in Table 8.


(11) $$Channel_{it}=\beta_{0}+\beta_{1}IC_{it}+\theta X_{it}+\mu_{i}+\nu_{t}+\varepsilon_{it}$$

**Table 8:** The results of channel analysis.

	Positive external channels		Negative external channels	
	Technological innovation effect	Resource allocation effect	Energy consumption effect	Population crowding effect
	(1)	(2)	(3)	(4)
*IC*	−11.439**	−0.068*	0.088**	0.009
	(−1.981)	(−1.823)	(2.006)	(0.346)
Controls	Yes	Yes	Yes	Yes
City fixed effect	Yes	Yes	Yes	Yes
Year fixed effect	Yes	Yes	Yes	Yes
Number of cities	285	285	285	285
Observations	4,558	4,560	4,560	4,555
*R*-squared	0.167	0.869	0.888	0.061

***p < 0.01, **p < 0.05, *p < 0.1. Robust *T*-statistics in parentheses.

Here *Channel_it_* denotes the channel variables that industrial co-agglomeration affects urban carbon emissions.

According to the realistic foundation of the empirical regressions, this paper decomposes the positive externalities of *IC* into a technological innovation path and resource allocation path. The negative externalities of *IC* are decomposed into an energy consumption path and a population crowding path. The coefficients in columns (1, 2) are both significantly negative, whereas the coefficient in columns (3, 4) are opposite. That is, *IC* can significantly stifle technological innovation and reduce resource allocation efficiency, and it may result in high energy consumption and excessive population concentration. These estimations indicate that the positive externalities brought about by *IC* are insufficient to mitigate negative environmental impacts, consistent with other findings (37, 63). This is mainly because the enterprises in the agglomeration area may hinder the entry of new enterprises for their own development. This phenomenon can lead to the difficulty of high-end innovation and makes them fall into the dilemma of inherent knowledge redundancy and new knowledge insufficiency, which exacerbates the negative externality of industrial agglomeration. It can be inferred that the positive externalities of *IC* are suppressed at a certain stage of development and negative externalities were common. It is crucial to consider the impact of *IC* on environmental pollution and environmental governance.

### 6.3. Further analysis in the mechanism of action

Based on mechanism analysis, market forces, and government intervention often play a moderating role in *IC* influencing *CE*. Therefore, this paper further analyzes the underlying associations between *IC* and *CE* from these two aspects, and the constructed model as shown in Equation. 12:


(12) $$CE_{it}=\beta_{0}+\beta_{1}IC_{it}+\beta_{2}IC_{it}\cdot M_{it}+\theta X_{it}+\mu_{i}+\nu_{t}+\varepsilon_{it}$$

where *M_it_* denotes the mechanism variables, namely the interaction term of *IC* and *ML* and the interaction term of *IC* and *GI*.

As for the coefficients of the interaction term in Table 9, the *GI* and *MC* both play a positive moderating role in the nexus between *IC* and *TCE*; nonetheless, they both have an opposite effect on the interaction between *IC* and *CEI*. The possible reasons lie in the following. First, excessive government intervention in *IC* is widespread in China, which leads to competition with the pursuit of policy rents in the agglomeration area and dramatically stimulates the *TCE* level. This result can also be confirmed in the study of Hong et al. (12). Moreover, the improvement of the marketization degree is not accompanied by the corresponding strength of environmental regulation due to imperfect market mechanisms. This makes the industries ignore the environmental protection problems during high-speed economic development. Second, marketization and government intervention can both be viewed as essential paths for high-quality economic development in the new normal. Their dynamic corporation is beneficial to relieve the pressure of *CEI via* the promotion of effective allocation of resources and the improvement in economic development efficiency.

**Table 9:** The results of mechanism analysis.

	*TCE*		*CEI*	
	Government intervention effect	Marketization level effect	Government intervention effect	Marketization level effect
	(1)	(2)	(3)	(4)
IC*GI	23.851***		−8.595**	
	(2.702)		(−2.162)	
IC*ML		1.014**		−0.727***
		(2.149)		(−3.314)
Controls	Yes	Yes	Yes	Yes
City fixed effect	Yes	Yes	Yes	Yes
Year fixed effect	Yes	Yes	Yes	Yes
Number of cities	285	285	285	285
Observations	4,560	4,560	4,560	4,560
*R*-squared	0.504	0.504	0.548	0.553

***p < 0.01, **p < 0.05, *p < 0.1. Robust *T*-statistics in parentheses.

## 7. Conclusion, policy implementations and future work

This paper conducts an empirical analysis of how industrial co-agglomeration affects urban carbon emissions through a series of econometric models, with the utilization of panel data from 285 Chinese cities at the prefecture level and above from 2005 to 2020. The following key points can be concluded.

Industrial co-agglomeration can intensify urban carbon emissions, both from the point of view of total carbon emissions and carbon emission intensity, and these aggravated effects are uneven and imbalanced. The presence of threshold effect is verified, namely, the inhibition effect of industrial co-agglomeration on total carbon emissions gradually weakened and the exacerbating influences are progressively significant with high-quality economic growth. By contrast, opposite trends were observed for carbon emission intensity.

The positive spillover, temporal lag, and spatial–temporal effects of urban carbon emissions within regions in China are indicated according to the spatial empirical results. From the overall results of effect decomposition, industrial co-agglomeration can stimulate total carbon emissions in neighboring and whole regions, and its cyclic accumulative impacts are valid in the long term, which provides evidence for the behavior of beggar-thy-neighbor. By contrast, industrial co-agglomeration intensifies the local carbon emission intensity, but it has no statistically significant influence on surrounding areas. Specifically, the above conclusions still hold after the consideration of endogeneity and robustness.

In addition, the heterogeneous impacts of industrial co-agglomeration on carbon emissions are supported, and positive spillover effects are more significant in the subsample of western, resource-oriented, and non-low-carbon pilot cities. In comparison, labor and land costs are the main factors influencing the relationship between industrial agglomeration and carbon emissions, rather than transaction costs. This paper also shows that the negative externalities of industrial co-agglomeration occupy a dominant position in high-quality economic development. The energy consumption effect and population crowding effect are the main reasons for the intensification of urban carbon emissions. Therefore, government intervention and marketization have different moderating effects on the selected carbon emission proxies. Moderate corporation and dynamic balance of these mechanisms are vital to the low-carbon economic development in the new status.

The following insights are provided for policymakers. First, local government should focus on the new development concept of a community with a shared future for mankind and avoid beggar-thy-neighbor patterns of industrial activities and environmental governance while promoting the low-carbon transformation of local enterprises. Local governments should fully consider the unique characteristics and strengths of different industries to prevent low-end, imitation, and homogenization behaviors and relieve local carbon emission pressures. The formulation of appropriate supporting policies and reasonable environmental regulation standards also contribute to avoiding the vicious competitive behaviors caused by policy rent behaviors among industries. Hence, under the guidance of a win-win development goal, governments should facilitate the extension of the regional industrial chain and establish a green industrial system among regions and promote the realization of the national carbon decoupling process.

Second, the targeted policies based on the geographical characteristics and economic attributes of different regions should be formulated. The government can cultivate differentiated leading industries and the specific industrial co-agglomeration mode based on local function orientations and development situations. For instance, the eastern regions should take advantage of its developed economy and geographical features and actively explore the industrial low-carbon management pattern *via* constant technological innovation and the exploitation of clean energy, such as coastal wind resources and nuclear power. The green and efficient development of resource-oriented cities has become crucial for the achievement of the double carbon goal, so governments should strive to improve energy consumption structure and low-carbon technology. The scope of low-carbon pilot cities should be properly expanded, which is conducive to stimulating the innovation vitality of cities and accelerating the low-carbon transformation of the economy.

Third, the government should seek a dynamic balance with marketization, to build a national unified market, and further strengthen the positive external effects of industrial co-agglomerations. From one aspect, the government should actively implement moderate policies and regulations to restrict, encourage, and supervise the development of industrial co-agglomeration. This is beneficial to the optimization and adjustment of the internal structure of the industries. From another view, it is essential to build a national unified market, which can not only accelerate the free flow of resource elements among regions but also promote resource exchange and information sharing among industries. This is also conducive to breaking the technological lock-in effect and promoting the effective exertion of the positive externalities of industrial co-agglomeration. To better grasp the balance between government intervention and market competition, promoting the combination of a promising government and an effective market is vital to achieving double carbon.

Although this study provides some bright empirical results and contributes to the existing literature, there are still some limitations that need to be further investigated in the future. Firstly, this paper only uses the geographic inverse distance matrix to explore the spatial effect, it is necessary to adopt the economic distance matrix, economic geography nested matrix, and other different types of spatial weight matrices to comprehensively explore the spatial effect of industrial co-agglomeration in the future studies. Moreover, the space–time weight matrix can also be considered to investigate the temporal and spatial effects of industrial agglomeration simultaneously. Secondly, this paper only studies the nexus between industrial co-agglomeration and carbon emissions, but the sources of urban environmental pollution are relatively complex. In detail, the industrial discharged wastewater, industrial smoke dust, nitrogen oxide, industrial sulfur dioxide, and other pollutant emission indicators should be fully considered to explore the environmental impact of industrial co-agglomeration. Thirdly, due to the limitation of data sources, this paper only considers the data from 2005 to 2020. In the future, the data period should be extended to 2022 to provide more detailed and accurate suggestions for the development of industrial co-agglomeration subject to data availability.

## Data availability statement

The original contributions presented in the study are included in the article/Supplementary material, further inquiries can be directed to the corresponding author.

## Author contributions

QS: conceptualization, methodology, and formal analysis. YP: data curation and writing—original draft, visualization, and investigation. YF: writing—review and editing, supervision, and resources. All authors contributed to the article and approved the submitted version.

## Funding

This research was supported by the youth program of the high-end science and technology innovation think tank of the Chinese Association for Science and Technology (grant no. 2021ZZZLFZB1207131), the Program for Science & Technology Innovation Talents in the Universities of the Henan Province (grant no. 2021-CX-018), and the Postdoctoral Research Foundation of China (grant no. 2022M720131).

## Conflict of interest

The authors declare that the research was conducted in the absence of any commercial or financial relationships that could be construed as a potential conflict of interest.

## Publisher’s note

All claims expressed in this article are solely those of the authors and do not necessarily represent those of their affiliated organizations, or those of the publisher, the editors and the reviewers. Any product that may be evaluated in this article, or claim that may be made by its manufacturer, is not guaranteed or endorsed by the publisher.
