# Supplementary material for: Identifying impacts of industrial co-agglomeration on carbon emissions: Evidence from China
Source: Front Public Health. 2023 Mar 23;11:1154729. doi: 10.3389/fpubh.2023.1154729 (PMC10076784; doi:10.3389/fpubh.2023.1154729)
Supplement: Supplementary file 1 [file Table_1.docx]

**Supplementary Materials**

1. Empirical Results and Analysis
2. Empirical Analysis of Baseline Regression

According to Table A.1, it is clear that the results of the mathematical model only with one constant also confirms the dramatic stimulating effect on urban carbon emissions, which is in line with the obtained highlights based on Eq. (1).

Table A.1. Estimation Results of *OLS* without Constant

| Core Variables | *TCE* | *CEI* |
| --- | --- | --- |
|  | (1) | (2) |
| *IC* | 14.828*** | 12.926*** |
|  | (12.537) | (35.768) |
| Controls | Yes | Yes |
| Observations | 4,560 | 4,560 |
| R-squared | 0.658 | 0.620 |
| F-value | 1756 | 1486 |

Notes: **** p<0.01, ** p<0.05, * p<0.1. T*-values in parentheses.

1. Empirical Analysis of Panel Quantile Regression

The panel quantile regression framework is involved to further investigates the nexus between *IC* and carbon emissions due to the distributional heterogeneity. The results of constructed models are summarized in Table A.2. For reflect the quantile model results in more detail, the plots of the regression on specific carbon emissions metrics at the 95% confidence level are shown in Fig. A.1. It is evident that *IC* exerts the significant positive influence on both *TCE* and *CEI* at all different quantiles, specifically the uneven effects are confirmed. Namely the intensification effect of *IC* on *TCE* shows an increasing fluctuation trend with the accumulation of carbon emission levels, nevertheless, the promotion effect of *IC* on *CEI* is characterized by continuous and stable increase.

The causal role of this case can be partially explained by the excessive population agglomeration. Local governments strongly advocate a new path of integrated and coordinated development of industries and talents at the early stage of agglomeration development, which tend to implement the "high-level talent introduction strategy" and "talent reserve strategy", so as to create a virtuous cycle of regional economic development. This representative policy-oriented action has led to mass migration flows and labor agglomeration, while the issue of overpopulation has gradually emerged. Overpopulation may result in the increase of household garbage, exhaust gas emissions and energy consumption, which limits on optimal critical value of regional development and environmental carrying capacity. Therefore, the aggravated and uneven influences of *IC* on urban carbon emissions can be supported evidently. As for a series of control variables, their differentiated effects are obvious.

**Table A.2.** Estimation Results of Panel Quantile Regression

| Core Variables | 10th | | 30th | | 50th | | 70th | | 90th | |
| --- | --- | --- | --- | --- | --- | --- | --- | --- | --- | --- |
|  | *TCE* | *CEI* | *TCE* | *CEI* | *TCE* | *CEI* | *TCE* | *CEI* | *TCE* | *CEI* |
|  | (1) | (2) | (3) | (4) | (5) | (6) | (7) | (8) | (9) | (10) |
| *IC* | 10.079*** | 1.552*** | 7.750*** | 2.394*** | 4.867*** | 2.448*** | 7.110*** | 4.128*** | 10.438** | 5.936*** |
|  | (12.824) | (6.834) | (5.625) | (5.409) | (4.104) | (7.218) | (3.951) | (8.584) | (2.133) | (5.531) |
| *FD* | -0.719*** | -0.131*** | -1.779*** | -0.403*** | -2.724*** | -0.524*** | -3.059*** | -0.487*** | -2.399*** | -0.798*** |
|  | (-6.724) | (-5.701) | (-7.888) | (-9.602) | (-12.120) | (-9.570) | (-8.800) | (-5.588) | (-6.834) | (-8.298) |
| *IU* | 0.583*** | 0.143*** | 3.257*** | 0.905*** | 4.659*** | 1.032*** | 7.431*** | 1.159*** | 4.614** | 1.749*** |
|  | (2.713) | (3.017) | (3.820) | (5.231) | (5.708) | (7.953) | (7.072) | (5.773) | (2.164) | (3.426) |
| *UR* | 7.230*** | -6.964*** | 13.849*** | -9.149*** | 23.983*** | -11.625*** | 33.978*** | -15.720*** | 63.112*** | -24.212*** |
|  | (6.838) | (-15.657) | (6.691) | (-14.332) | (7.774) | (-20.223) | (9.336) | (-26.903) | (8.158) | (-23.802) |
| *FDI* | 36.414*** | 8.924*** | 51.209*** | 7.230*** | 56.294*** | 2.633 | 93.387*** | 1.970 | 249.952*** | 2.437 |
|  | (6.081) | (3.748) | (3.577) | (2.812) | (4.586) | (0.985) | (4.332) | (0.238) | (3.042) | (0.270) |
| Constant | -1.682** | 5.179*** | 3.545*** | 7.884*** | 9.362*** | 11.308*** | 8.990*** | 14.652*** | 8.292** | 23.736*** |
|  | (-2.203) | (15.561) | (2.584) | (12.292) | (5.696) | (21.835) | (3.448) | (28.894) | (2.001) | (15.288) |
| Observations | 4,560 | 4,560 | 4,560 | 4,560 | 4,560 | 4,560 | 4,560 | 4,560 | 4,560 | 5,130 |
| Number of cities | 285 | 285 | 285 | 285 | 285 | 285 | 285 | 285 | 285 | 285 |

Notes: **** p<0.01, ** p<0.05, * p<0.1. T*-values in parentheses.

| 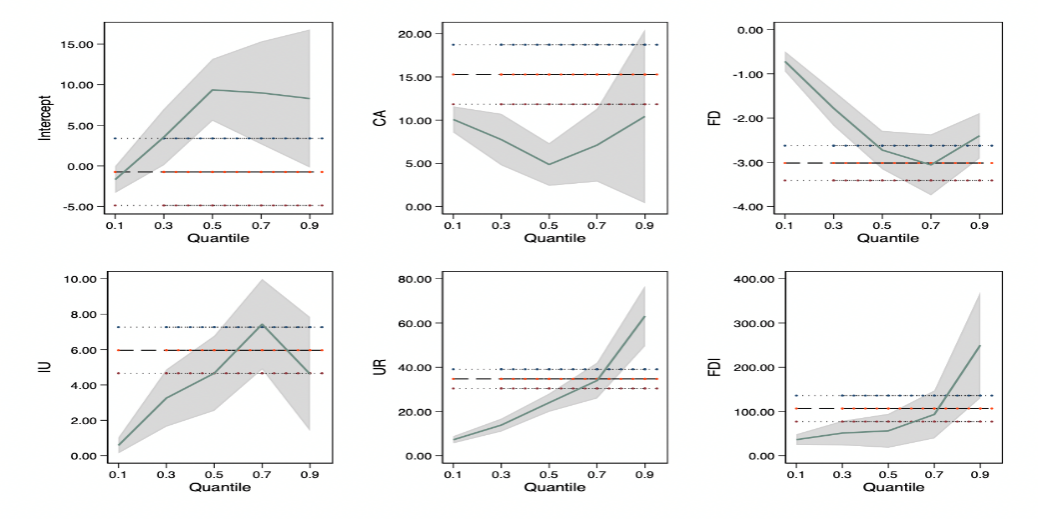 | 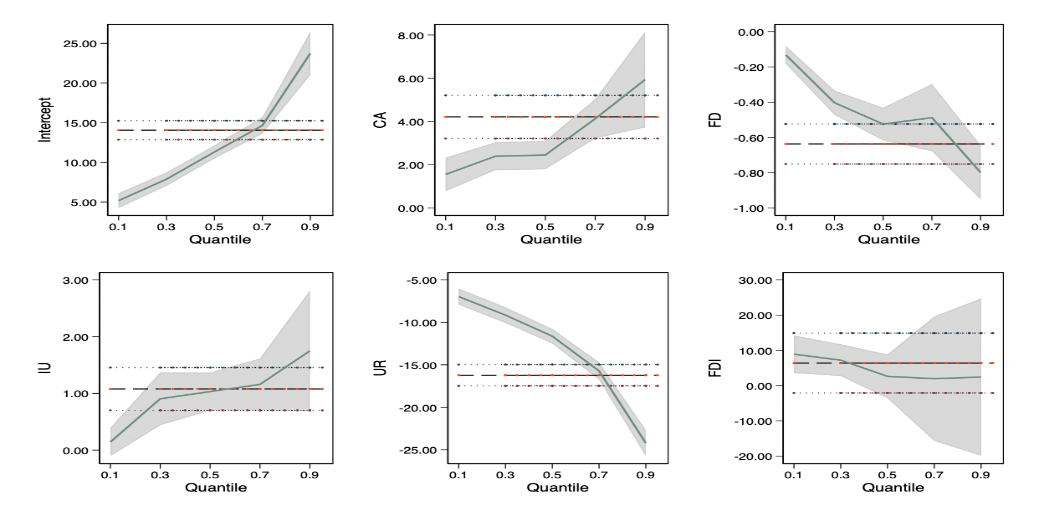 |
| --- | --- |
| (a) Plot of quantile regression of *TCE* | (b) Plot of quantile regression of *CEI* |
| **Fig. A.1.** Quantile Regression Coefficient Change Graph with 95% Confidence Level. | |

1. Empirical Analysis of Panel Threshold Model

This subsection applies the panel threshold model to conduct in-depth research of the non-linear nexus under different economic development levels, which are characterized in the form of *PGDP*. Firstly, the existence test of the threshold effect should be explored and results are presented in Table A.3. The single threshold model and double threshold model are appropriate at 1% significance level when adopting *TCE* and *CEI* as measurement metrics respectively, which confirm the threshold effect of *IC* on carbon emissions. Subsequently, the detailed estimated results are reported in Table A.4. and Fig. A.2. to Fig. A.3. present the likelihood ratio statistic tests of these panel threshold model.

In terms of *TCE*, *IC* plays significant negative role on *TCE* at the 1% confidence level when the level of PGDP is below the value of 10.311, nonetheless, its emission-reduction effect is gradually weakened that has turned into the forward direction if *PGDP* level exceeds this threshold value. Absolutely, the empirical results provide evidence that *IC* moderately curbs *TCE* in the start-up phase of regional economic development, while the inhibitory effects have been transformed as the rapid economic development. The possible reasons for this phenomenon are that the positive externalities of *IC* play the dominant position on carbon emission reduction at the initial phase of economic expansion, which can improve the utilization efficiency of resource through information sharing, advanced management concept dissemination, technology spillover effect and other channels, so as to achieve the short-term goal of carbon emission control. By contrast, the crowding-out effect and path-dependent effect gradually show up in the process of economic development, which not only results in the homogenization and vicious competition, but also impede technological diffusion and innovation. This is the vital reason to limit the optimal critical value of co-agglomeration and reduce the regional energy use efficiency, which poses adverse shocks to the intensification of urban *CE* degree.

As for the *CEI*, it can be observed that the *IC* has the persistent aggravated effect on it before *PGDP* reaches the second threshold value of 9.438, nevertheless, this positive promoting effect gradually reverses and becomes less significant. Indeed, this is the specific difference between the impact of *IC* on *TCE* and *CEI* due to the consideration of economic factors in *CEI*. The reasonable explanation relies on that the local government has gradually realized the importance of corporation between high-quality economic development and ecological environment protection, which is more proactive in innovating low-carbon development paths. For instance, *IC* has gradually realized the recycling of products and resource with the promotion of the third-party pollution control mechanism. Meanwhile tapping the potential of environmental infrastructure to control carbon emissions has gradually become the crucial path of pollutants control in industrial agglomeration regions. Hence, with the of China's rapid industrial economy development, the evolution trend of *TCE* and *CEI* is not consistent to some extent, there is still a long way to go to achieve the targets of the dual-carbon goal.

On the whole, the formation of industrial agglomeration area is based on the current situation of economic development, namely the higher economic level can attract more industries and enterprises to locate in which contributes to yield the nonlinear nexus between industrial co-agglomeration and urban carbon emissions. Thereby, the regression results provide empirical evidence for the threshold effect.

**Table A.3.** Threshold Effect Significant Test

| Explained  Variables | Model | F-value | P-value | Critical Value | | | Threshold  Estimators | 95%  Confidence  Interval |
| --- | --- | --- | --- | --- | --- | --- | --- | --- |
|  |  |  |  | 10% | 5% | 1% |  |  |
| *TCE* | Single threshold | 214.51*** | 0.000 | 80.025 | 93.375 | 139.485 | 10.311 | [10.283, 10.354] |
|  | Double threshold | 52.71 | 0.334 | 81.089 | 97.790 | 150.866 | 10.354 | [10.297, 10.390] |
|  |  |  |  |  |  |  | 9.969 | [9.948, 9.989] |
|  | Triple threshold | 48.54 | 0.570 | 103.085 | 129.166 | 165.917 | 9.398 | [9.375, 9.411] |
| *CEI* | Single threshold | 260.65*** | 0.000 | 95.122 | 116.323 | 157.629 | 8.780 | [8.748, 8.808] |
|  | Double threshold | 186.88*** | 0.038 | 87.377 | 138.626 | 290.784 | 8.780 | [8.748, 8.808] |
|  |  |  |  |  |  |  | 9.438 | [9.424, 9.450] |
|  | Triple threshold | 90.36 | 0.750 | 346.787 | 422.469 | 487.178 | 8.997 | [8.973, 9.016] |

Notes: **** p<0.01, ** p<0.05, * p<0.1.*

**Table A.4.** Panel threshold model results for the carbon emission intensity

| Core Variables | *TCE* | | | *CEI* | | |
| --- | --- | --- | --- | --- | --- | --- |
|  | Single Threshold | Double Threshold | Triple Threshold | Single Threshold | Double Threshold | Triple Threshold |
|  | (1) | (2) | (4) | (5) | (6) | (7) |
| *FD* | 0.106 | 0.116 | 0.086 | 0.098* | 0.133*** | 0.153*** |
|  | (1.085) | (1.191) | (0.889) | (1.883) | (2.616) | (3.026) |
| *IU* | 0.031 | -0.130 | -0.211 | -0.831*** | -0.778*** | -0.778*** |
|  | (0.109) | (-0.462) | (-0.754) | (-5.551) | (-5.300) | (-5.350) |
| *UR* | 42.232*** | 40.756*** | 39.066*** | -22.803*** | -20.965*** | -19.995*** |
|  | (40.783) | (38.728) | (36.292) | (-40.626) | (-36.948) | (-34.990) |
| *FDI* | -28.961*** | -28.440*** | -30.739*** | -16.411*** | -14.271*** | -13.585*** |
|  | (-5.433) | (-5.363) | (-5.815) | (-5.808) | (-5.144) | (-4.943) |
| *IC*(*PGDP*≤10.311) | -2.903*** |  |  |  |  |  |
|  | (-3.651) |  |  |  |  |  |
| *IC*(*PGDP*>10.311) | 8.005*** |  |  |  |  |  |
|  | (7.996) |  |  |  |  |  |
| *IC*(*PGDP*≤8.780) |  |  |  | 7.008*** |  |  |
|  |  |  |  | (12.819) |  |  |
| *IC*(*PGDP*>8.780) |  |  |  | 1.069** |  |  |
|  |  |  |  | (2.573) |  |  |
| *IC*(*PGDP*≤9.969) |  | -3.449*** |  |  |  |  |
|  |  | (-4.331) |  |  |  |  |
| *IC* (9.969<*PGDP*≤10.354) |  | 0.975 |  |  |  |  |
|  |  | (1.042) |  |  |  |  |
| *IC*(*PGDP*> 10.354) |  | 11.854*** |  |  |  |  |
|  |  | (10.547) |  |  |  |  |
| *IC*(*PGDP*≤8.780) |  |  |  |  | 8.988*** |  |
|  |  |  |  |  | (16.158) |  |
| *IC*(8.780<*PGDP*≤9.438) |  |  |  |  | 2.806*** |  |
|  |  |  |  |  | (6.559) |  |
| *IC*(*PGDP*>9.438) |  |  |  |  | -0.421 |  |
|  |  |  |  |  | (-0.996) |  |
| *IC*(*PGDP*≤9.398) |  |  | -5.342*** |  |  |  |
|  |  |  | (-6.356) |  |  |  |
| *IC*(9.398<*PGDP*≤9.969) |  |  | -2.139*** |  |  |  |
|  |  |  | (-2.622) |  |  |  |
| *IC*(9.969<*PGDP*≤10.354) |  |  | 2.444** |  |  |  |
|  |  |  | (2.559) |  |  |  |
| *IC*(*PGDP*> 10.354) |  |  | 13.296*** |  |  |  |
|  |  |  | (11.680) |  |  |  |
| *IC*(*PGDP*≤8.780) |  |  |  |  |  | 10.652*** |
|  |  |  |  |  |  | (18.390) |
| *IC*(8.780<*PGDP*≤8.997) |  |  |  |  |  | 4.890*** |
|  |  |  |  |  |  | (10.201) |
| *IC*(8.997<*PGDP*≤9.438) |  |  |  |  |  | 2.331*** |
|  |  |  |  |  |  | (5.464) |
| *IC*(*PGDP*>9.438) |  |  |  |  |  | -0.942** |
|  |  |  |  |  |  | (-2.233) |
| Constant | 7.125*** | 7.633*** | 8.621*** | 19.591*** | 18.562*** | 18.087*** |
|  | (8.776) | (9.392) | (10.492) | (45.239) | (43.014) | (42.030) |
| R-squared | 0.358 | 0.365 | 0.371 | 0.406 | 0.429 | 0.440 |
| Number of cities | 285 | 285 | 285 | 285 | 285 | 285 |
| Observations | 4,560 | 4,560 | 4,560 | 4,560 | 4,560 | 4,560 |

Notes: **** p<0.01, ** p<0.05, * p<0.1. T*-values in parentheses.


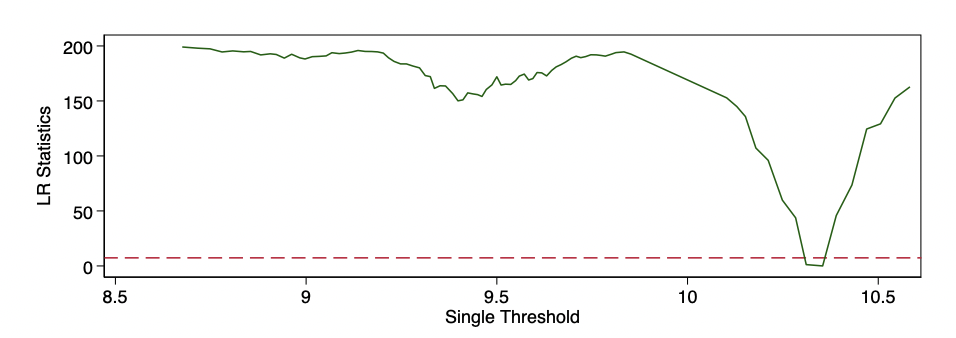


Fig. A.2. Likelihood Ratio Statistic Diagram of *TCE*.

| 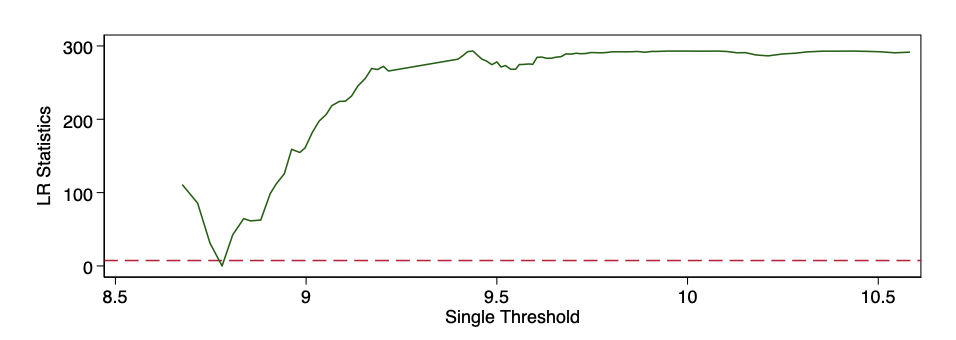 | 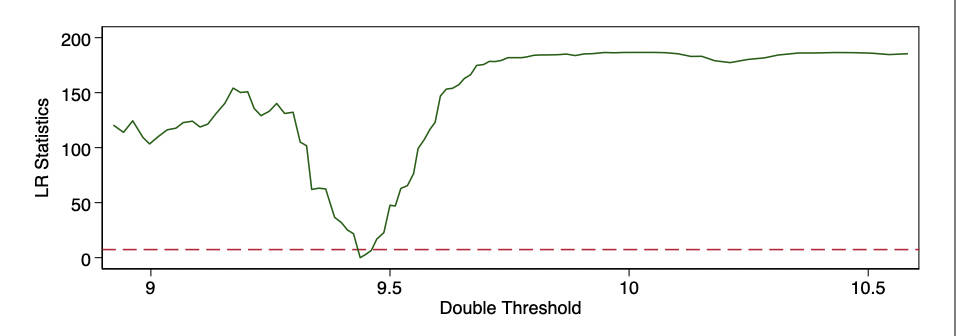 |
| --- | --- |
| (a) Single threshold model | (b) Double threshold model |
| **Fig. A.3.** Likelihood Ratio Statistic Diagram of *CEI*. | |

- 1. Empirical Analysis of Static SDM
     1. Spatial Correlation and Suitability Test

According to Table B.1, it is clear that Moran’s I and Geary’s C of carbon emissions in 2005-2020 all pass the significant test at 1% confidence level, which verifies the validity of the application of spatial econometric models. Besides, the statistic values of Moran’s I are all exceed the zero and the Geary’s C are all lower than one, revealing urban *CE* has significant positive spatial agglomeration. And the values of proxies indicate that the spatial auto-correlation fluctuates over time, especially the auto-correlation degree of IC between regions is continually strengthen. This paper also draws some crucial scatterplots of Maran’s I of carbon emissions in Fig. B.1. According to the graphs and table, spatial distribution of *IC* and *CE* in China in majority of cities are distributed in the first and third quadrants (H-H and L-L aggregation zones), which provides evidence for the positive spatial auto-correlations in sample period.

**Table B.1.** Results of Moran’s I and Geary’s C

| Year | Moran’s I | | | Geary’s C | | |
| --- | --- | --- | --- | --- | --- | --- |
|  | *IC* | *TCE* | *CEI* | *IC* | *TCE* | *CEI* |
|  | (1) | (2) | (3) | (4) | (5) | (6) |
| 2005 | 0.182*** | 0.273*** | 0.191*** | 0.795*** | 0.620*** | 0.703*** |
|  | (3.625) | (5.682) | (3.896) | (-3.708) | (-3.985) | (-3.733) |
| 2006 | 0.177*** | 0.280*** | 0.189*** | 0.791*** | 0.618*** | 0.703*** |
|  | (3.538) | (5.816) | (3.867) | (-3.778) | (-4.004) | (-3.677) |
| 2007 | 0.128*** | 0.298*** | 0.185*** | 0.846*** | 0.607*** | 0.712*** |
|  | (2.584) | (6.152) | (3.786) | (-2.788) | (-4.349) | (-3.597) |
| 2008 | 0.134*** | 0.310*** | 0.180*** | 0.847*** | 0.600*** | 0.725*** |
|  | (2.696) | (6.354) | (3.679) | (-2.766) | (-4.607) | (-3.612) |
| 2009 | 0.166*** | 0.297*** | 0.210*** | 0.819*** | 0.610*** | 0.692*** |
|  | (3.328) | (6.111) | (4.245) | (-3.266) | (-4.391) | (-4.353) |
| 2010 | 0.144*** | 0.300*** | 0.222*** | 0.827*** | 0.611*** | 0.678*** |
|  | (2.881) | (6.143) | (4.469) | (-3.128) | (-4.499) | (-4.596) |
| 2011 | 0.194*** | 0.313*** | 0.241*** | 0.775*** | 0.615*** | 0.664*** |
|  | (4.864) | (6.344) | (4.843) | (-3.983) | (-4.958) | (-5.001) |
| 2012 | 0.203*** | 0.311*** | 0.243*** | 0.773*** | 0.617*** | 0.661*** |
|  | (4.049) | (6.307) | (4.884) | (-4.067) | (-4.897) | (-5.046) |
| 2013 | 0.193*** | 0.302*** | 0.267*** | 0.789*** | 0.638*** | 0.645*** |
|  | (3.851) | (6.097) | (5.359) | (-3.802) | (-4.893) | (-5.278) |
| 2014 | 0.135*** | 0.298*** | 0.256*** | 0.863*** | 0.642*** | 0.654*** |
|  | (2.706) | (6.016) | (5.129) | (-2.486) | (-4.834) | (-5.190) |
| 2015 | 0.131*** | 0.310*** | 0.272*** | 0.885*** | 0.633*** | 0.638*** |
|  | (2.641) | (6.248) | (5.446) | (-2.101) | (-5.063) | (-5.540) |
| 2016 | 0.129*** | 0.309*** | 0.273*** | 0.865*** | 0.634*** | 0.641*** |
|  | (2.593) | (6.218) | (5.464) | (-2.458) | (-5.042) | (-5.531) |
| 2017 | 0.204*** | 0.292*** | 0.274*** | 0.779*** | 0.652*** | 0.627*** |
|  | (4.069) | (5.884) | (5.488) | (-4.014) | (-4.813) | (-5.655) |
| 2018 | 0.207*** | 0.278*** | 0.277*** | 0.774*** | 0.668*** | 0.628*** |
|  | (4.116) | (5.594) | (5.544) | (-4.094) | (-4.587) | (-5.645) |
| 2019 | 0.223*** | 0.265*** | 0.334*** | 0.749*** | 0.684*** | 0.580*** |
|  | (4.435) | (5.353) | (6.681) | (-4.546) | (-4.370) | (-6.327) |
| 2020 | 0.223*** | 0.256*** | 0.310*** | 0.749*** | 0.697*** | 0.606*** |
|  | (4.435) | (5.162) | (6.195) | (-4.546) | (-4.167) | (-6.032) |

Notes: **** p<0.01, ** p<0.05, * p<0.1. Z* -values in parentheses.

|  |  |
| --- | --- |
| (a) Plot Diagram of Moran’s I in *TCE* | |
|  |  |
| (b) Plot Diagram of Moran’s I in *CEI* | |

**Fig. B.1.** Scatter Diagram of Moran’s I in Sample Cities during 2005 and 2020.

- - 1. Empirical Analysis of Spatial Durbin Model

The regression results are represented in the Table B.2. According to the estimations, the spatial autoregressive coefficients of two carbon emission measurement proxies are all exceed zero and are significant at 1% confidence interval, which show that the carbon emissions of sample cities in China have obvious positive spatial spillover effect. Namely the local carbon emissions level can be dramatically affected by the pollutant emissions in the geographically adjacent cities. According to the general regression coefficients and spatial regression coefficients, it is evident that *IC* can significantly promote the regional *CEI* and the *IC* of neighboring regions exert the intensifying effect on regional *TCE*.

These empirical results demonstrate the following crucial points: firstly, the development within the region needs to transform the crude economic development mode to the high-quality green economic development, which should pay more attention to properly handle the relationship between "development" and "ecology", so as to efficiently restrain the local *CEI* levels; secondly, the beggar-thy-neighbor practices by governments are needed to be further consideration, although this policy action can eliminate local environmental stress to some extent, it has no beneficial effect on the ecological environment of the whole society and the country, while it may also accumulate regional *TCE* level via spatial feedback effect. Besides, it is undoubted that the differentiated effects of control variables are confirmed based on the regression estimations.

Based on the above theoretical analysis, this paper also decomposes the overflow effect of *SDM* into direct effect, indirect effect, and total effect. The coefficient of indirect effect of *TCE* and the direct effect of *CEI* are both significant positive at 1% significance level, which also verify the above key points. In general, *IC* can dramatically aggravate urban *TCE* level that is supported by the significance of total effect, and it also have a tendency to intensify urban *CEI*. Moreover, different control variables have diversified influence mechanisms on two carbon emission indicators, which reflects the complexity of the economic factors on the formulation of carbon emissions policies and reveals the challenges in achieving the transition from peak carbon to carbon neutral are undoubtedly enormous.

**Table B.2.** Results of SDM with Dual-fixed effects

| Explained  Variables | Core  Variables | Main | Weight Metrix | Direct Effect | Indirect Effect | Total Effect |
| --- | --- | --- | --- | --- | --- | --- |
|  |  | (1) | (2) | (3) | (4) | (5) |
| *TCE* | *IC* | -0.192 | 2.533*** | 0.175 | 3.521*** | 3.695** |
|  |  | (-0.303) | (2.689) | (0.260) | (2.741) | (2.289) |
|  | *FD* | -0.275*** | 0.367*** | -0.241*** | 0.382** | 0.140 |
|  |  | (-3.093) | (2.633) | (-2.850) | (2.073) | (0.680) |
|  | *IU* | -0.590* | 2.185*** | -0.289 | 2.742*** | 2.453*** |
|  |  | (-1.910) | (4.717) | (-0.982) | (4.790) | (3.731) |
|  | *UR* | 10.106*** | -0.991 | 10.467*** | 3.704 | 14.171*** |
|  |  | (7.811) | (-0.547) | (8.185) | (1.566) | (4.996) |
|  | *FDI* | -22.955*** | 21.744*** | -20.987*** | 19.526** | -1.461 |
|  |  | (-4.748) | (3.236) | (-4.545) | (2.228) | (-0.148) |
|  | ρ |  | 0.348*** |  |  |  |
|  |  |  | (24.202) |  |  |  |
|  | R-squared | 0.301 | 0.301 | 0.301 | 0.301 | 0.301 |
| *CEI* | *IC* | 1.397*** | -0.652 | 1.384*** | -0.369 | 1.015 |
|  |  | (4.044) | (-1.264) | (3.866) | (-0.608) | (1.359) |
|  | *FD* | 0.113** | 0.321*** | 0.141*** | 0.422*** | 0.562*** |
|  |  | (2.336) | (4.196) | (3.076) | (4.751) | (5.900) |
|  | *IU* | 0.807*** | -0.437* | 0.803*** | -0.320 | 0.483 |
|  |  | (4.769) | (-1.723) | (5.067) | (-1.161) | (1.592) |
|  | *UR* | -1.577** | -4.500*** | -1.979*** | -5.808*** | -7.788*** |
|  |  | (-2.223) | (-4.541) | (-2.865) | (-5.097) | (-5.858) |
|  | *FDI* | -11.952*** | -21.894*** | -13.978*** | -29.724*** | -43.702*** |
|  |  | (-4.511) | (-5.931) | (-5.578) | (-6.941) | (-9.547) |
|  | ρ |  | 0.229*** |  |  |  |
|  |  |  | (13.474) |  |  |  |
|  | R-squared | 0.245 | 0.245 | 0.245 | 0.245 | 0.245 |
| - | Observations | 4,560 | 4,560 | 4,560 | 4,560 | 4,560 |
| - | Number of cities | 285 | 285 | 285 | 285 | 285 |

Notes: **** p<0.01, ** p<0.05, * p<0.1. Z-*values in parentheses.

1. Robustness Test

To further confirm the validity of the empirical results, this paper conducts the robustness tests from diversified perspectives, including replacing explained variable, replacing research samples, and constructing new spatial weight matrices. The re-regression results are shown in Table C.1.

1. Replace Explained Variable

Consider that China is the most populous nation and the second largest economy in the world as so far, it is essential to takes the demographic factor into consideration of the carbon emission measurement. This subsection adopts carbon emissions per capita (*PCE*) as a suitable proxy to quantify the carbon emission levels among different regions. Based on the re-regression results, it is obvious that *IC* can also accelerate *PCE* to some extent, which confirms the intensified effects of *IC* on urban carbon emissions and the robustness of the baseline regression.

1. Replace Sample

Different cities are in several different stages with distinct phased goals, which also have distinct high-tech technology levels, resource endowment, industrial structure and other characteristics. Especially the provincial capital cities that have more favorable features when comparing with the prefecture-level cities. Thus, this subsection conducts dynamic *SDM* based on new sample sizes which excludes this type of cities respectively. The provincial capital cities cover 26 cities such as Zhengzhou, Hangzhou, Chengdu, Xian, Guangzhou, Hefei etc. According to the re-regression estimations, the positive spatial spillover effect of carbon emissions in China is verify again. Besides, the promotion effects of *IC* on urban carbon emissions are also clearly observed. The sign of the coefficients of rest parameters keeps consistent, only the level of significance changed. Indeed, the accumulative effect of *IC* on *TCE* and diminishing effect of *CEI* is also supported via the comparison between the absolute values of the short-term effects and long-term effects, which also proves the robustness of the baseline results.

1. Replace Spatial Weight Matrix

Considering the regional diffusion process of industrial factors and the spatial correlation of industrial development can also appear among cities with similar economic attribute and geographically adjacent position, this paper also constructs the economic distance weight matrix and geographically adjacent matrix to investigate the associations between *IC* and urban *CE*. The constructed matrices are shown as Eq. (1) and Eq. (2). The re-regression estimations also prove the aggregated effect of *IC* on urban *CE* in China, specifically its accumulative effect on *TCE* and diminishing effect of *CEI* in long run are both supported again. The rest regression coefficients basically remain the same directions and there are only some changes in the significance level, which again evidently validates the robustness of benchmark estimations.

$\boldsymbol{W}_{\boldsymbol{ij}}^{\boldsymbol{1}}\boldsymbol{=}\frac{\boldsymbol{1}}{\left| \boldsymbol{pgd}\boldsymbol{p}_{\boldsymbol{i}}\boldsymbol{-pgd}\boldsymbol{p}_{\boldsymbol{j}} \right|}$ (1)

$W_{ij}^{2}=\left\{ \begin{aligned} 1, i and j are adjacent \\ 0, i and j are not adjacent \end{aligned} \right.$ (2)

where *pgdp_i_* and *pgdp_j_* is the average *PGDP* of sample period in city *i* and *j*; *W^1^_ij_* is the obtained economic distance between regions. *W^2^_ij_* represents the geographically adjacent matrix.

**Table C.1.** Results of Robustness Test

| Effects | Core Variables | Replace Explained Variable | Replace Sample | | | | Replace Weight Matrix | | | | | |  |
| --- | --- | --- | --- | --- | --- | --- | --- | --- | --- | --- | --- | --- | --- |
|  |  | Per Capita  Carbon Emission | Eliminate Provincial  Capital Cities | | | Economic Distance Weight Matrix | | | Geographical Adjacent Weight Matrix | | |  |  |
|  |  | *PCE* | *TCE* | *CEI* | *TCE* | | | *CEI* | | *TCE* | *CEI* | | |
|  |  | (1) | (2) | (3) | (4) | | | (5) | | (6) | (7) | | |
| Spatial  Effect | *IC* | 0.350 | 0.907 | 1.161*** | 0.192 | | | 0.998*** | | 1.127* | 1.538*** | | |
|  |  | (1.030) | (1.587) | (3.343) | (0.302) | | | (2.991) | | (1.819) | (4.955) | | |
|  | *W·IC* | 0.799 | 0.447 | -1.279** | 5.253*** | | | -3.227*** | | -0.426 | -1.740*** | | |
|  |  | (1.576) | (0.518) | (-2.440) | (2.849) | | | (-3.336) | | (-0.353) | (-2.875) | | |
|  | ρ | 0.113*** | 0.107*** | 0.082*** | 0.026 | | | 0.082** | | 0.124*** | 0.156*** | | |
|  |  | (6.763) | (4.391) | (3.330) | (0.652) | | | (2.105) | | (4.586) | (5.769) | | |
| Direct  Effect | Short-term | 0.370 | 0.904 | 1.114*** | 0.183 | | | 0.951*** | | 1.094* | 1.471*** | | |
|  |  | (1.129) | (1.638) | (3.323) | (0.298) | | | (2.939) | | (1.827) | (4.886) | | |
|  | Long-term | 0.549 | 1.128* | 1.024*** | 0.477 | | | 0.920*** | | 1.111* | 1.371*** | | |
|  |  | (1.485) | (1.734) | (2.889) | (0.742) | | | (2.827) | | (1.734) | (4.022) | | |
| Indirect  Effect | Short-term | 0.915 | 0.595 | -1.252** | 5.395*** | | | -3.397*** | | -0.311 | -1.721** | | |
|  |  | (1.623) | (0.622) | (-2.191) | (2.746) | | | (-3.097) | | (-0.224) | (-2.393) | | |
|  | Long-term | 1.710* | 1.851 | -1.224 | 8.589*** | | | -3.589*** | | 0.121 | -1.850 | | |
|  |  | (1.829) | (1.031) | (-1.576) | (2.717) | | | (-3.011) | | (0.057) | (-1.383) | | |
| - | Control variables | Yes | Yes | Yes | Yes | | | Yes | | Yes | Yes | | |
| - | Observations | 4,275 | 3,885 | 3,885 | 4,275 | | | 4,275 | | 4,275 | 4,275 | | |
| - | R-squared | 0.291 | 0.520 | 0.487 | 0.435 | | | 0.398 | | 0.499 | 0.596 | | |
| - | Number of cities | 285 | 259 | 259 | 285 | | | 285 | | 285 | 285 | | |

Notes: **** p<0.01, ** p<0.05, * p<0.1. Z*-values in parentheses.
